# Efficacy and safety of Gyejibokryeong-hwan (GBH) in major depressive disorder: study protocol for multicentre randomised controlled trial

**DOI:** 10.1186/s13063-022-06339-0

**Published:** 2022-06-01

**Authors:** Yujin Choi, In Chul Jung, Ju Yeon Kim, Seung-Hun Cho, Yunna Kim, Sun-Yong Chung, Hui-Yong Kwak, Doo Suk Lee, Wonwoo Lee, In-Jeong Nam, Changsop Yang, Mi Young Lee

**Affiliations:** 1 KM Science Research Division, Korea Institute of Oriental Medicine, Daejeon, Republic of Korea; 2 Department of Oriental Neuropsychiatry, College of Korean Medicine, Daejeon University, Daejeon, 34520 Republic of Korea; 3 Department of Neuropsychiatry, Daejeon Korean Medicine Hospital of Daejeon University, Daejeon, 35235 Republic of Korea; 4 Department of Neuropsychiatry, College of Korean Medicine, Kyung Hee University, Seoul, Republic of Korea; 5 Research group of Neuroscience, East-West Medical Research Institute, WHO Collaborating Center, Kyung Hee University, Seoul, Republic of Korea; 6 Department of Clinical Korean Medicine, Graduated School, Kyung Hee University, Seoul, Republic of Korea; 7 R&D Center for Innovative Medicines, Helixmith Co., Ltd., Seoul, Republic of Korea; 8 KM Convergence Research Division, Korea Institute of Oriental Medicine, Daejeon, Republic of Korea

**Keywords:** Major depressive disorder, Phase II study, *Gyejibokryeong-hwan*, *Guizhifuling-wan*, *Keishibukuryo-Gan*, Herbal medicine, Randomised controlled trial (RCT)

## Abstract

**Background:**

Gyejibokryeong-hwan (GBH) is an herbal medicine composed of five herbs. It has been widely used to treat gynaecological diseases in traditional East Asian medicine. Recent animal studies suggest antidepressant effects of GBH. In this trial, we explore the efficacy and safety of GBH in patients with major depressive disorder and to identify the optimal dose for the next phase III trial.

**Methods:**

This trial will enrol 126 patients diagnosed with major depressive disorder and not treated with antidepressants. Participants will be randomised to receive a high or a low dose of GBH or placebo granules. The study drugs will be administered three times a day, for 8 weeks. The 17-item Hamilton Depression Rating Scale (HDRS) will be used to measure the severity of depressive symptoms at weeks 2, 4, 6, 8, and 12. The primary efficacy endpoint is the change from baseline in HDRS-17 total score post-treatment at week 8. Analysis of covariance will be based on the baseline HDRS-17 total score and site as the covariates. Safety assessment will be based on the frequency of adverse events. The severity and causality of the study drug will be assessed.

**Discussion:**

This study is designed to evaluate the efficacy and safety of GBH granules compared with placebo in patients with major depressive disorder.

**Trial registration:**

Clinical Research Information Service KCT0004417. Registered on November 1, 2019 (prospective registration)

## Administrative information

Note: The numbers in curly brackets in this protocol refer to SPIRIT checklist items. (http://www.equator-network.org/reporting-guidelines/spirit-2013-statement-defining-standard-protocol-items-for-clinical-trials/).

**Table:** 

Title {1}	Efficacy and safety of Gyejibokryeong-hwan (GBH) in major depressive disorder: study protocol for multicentre randomised controlled trial
Trial registration {2a and 2b}	KCT0004417 Clinical Research Information Service (CRIS) https://cris.nih.go.kr/cris/search/detailSearch.do/18015 registered on November 1^st^, 2019
Protocol version {3}	Version: 2.8 Date: 2020.05.28
Funding {4}	This study is supported by grants from the Korea Institute of Oriental Medicine (KIOM) [KSN2021220, KSN20214271] and Traditional Korean Medicine R&D Project, Ministry of Health & Welfare, Republic of Korea [HI15C0006].
Author details {5a}	Yujin Choi, In Chul Jung, Ju Yeon Kim, Seung-Hun Cho, Yunna Kim, Sun-Yong Chung, Hui-Yong Kwak, Changsop Yang, Mi Young Lee
Name and contact information for the trial sponsor {5b}	Korea Institute of Oriental Medicine, Daejeon, Republic of Korea
Role of sponsor {5c}	As a government-funded research institute and clinical trial application holder, KIOM is responsible for the study design; the analyses, or interpretation of data; the writing of the manuscript, or the decision to publish the results.

## Introduction

### Background and rationale {6a}

Major depressive disorder (MDD) is one of the depressive disorders characterised by major depressive episodes, which are diagnosed by the presence of persistent depressed mood or anhedonia, and additional symptoms of disturbed appetite or sleep, psychomotor agitation or retardation, fatigue, feeling of worthlessness, decreased concentration, or suicidal thoughts for at least 2 weeks [1, 2]. In Korea, the reported prevalence of depression was 2.8% in 2002, and 5.3% in 2013 which has increased with time [3]. Also, the suicide rate in Korea was 24.6 per 100,000 person in 2019, which was the highest among the Organisation for Economic Cooperation and Development (OECD) countries [4]. Antidepressant monotherapy with escitalopram or sertraline is recommended as the first-line strategy for mild-to-moderate depressive disorders without psychotic features in Korea [5, 6]. Nonetheless, new medications are needed for rapid amelioration of depression with fewer side effects.

*Gyejibokryeong-hwan* (GBH, *Guizhifuling-wan* in Chinese and *Keishibukuryo-gan* in Japanese) is a representative herbal formulation of traditional East Asian medicine (TEAM). It is composed of *Cinnamomi Ramulus*, *Poria Sclerotium*, *Moutan Radicis Cortex*, *Persicae Semen*, and *Paeoniae Radix*. According to the TEAM theory, GBH is one of the herbal formulae used to treat “qi stagnation and blood stasis” syndrome. GBH has been widely used to treat various gynaecological and cardiovascular diseases, for example, primary dysmenorrhea [7], endometriosis [8], hot flashes [9], climacteric syndrome [10–13], and angina pectoris [14].

The Korea Institute of Oriental Medicine (KIOM) established the project to investigate the novel indication for ancient herbal medicines and extend the indications (“drug repositioning project of herbal medicine”). Based on in vitro and in vivo screening tests, GBH was selected as one of the best candidates for alleviating depressive symptoms. GBH showed antidepressant-like effects in mouse models of reserpine-induced depression [15]. Also, potential neuroprotective effects of GBH were reported in lipopolysaccharide-stimulated BV2 microglia [16]. We also conducted a prospective observational study of herbal medicine in patients with depressive disorder, and GBH was helpful to patients and showed clinical adaptability [17]. Also, few clinical studies reported the role of GBH in managing depressive symptoms. In the previous two clinical studies of patients with asymptomatic cerebral infarction conducted in Japan, treatment with GBH extract improved the scores on the self-rating depression scale [18]. Also, patients who were treated with GBH scored lower on the self-rating depression scale than those who were not [19]. The potential role of GBH in improving depressive symptoms in patients with cerebrovascular diseases was reported. Meanwhile, the role of GBH compared with placebo in patients with depressive disorders has yet to be elucidated. Animal and clinical studies suggested the possible effects of GBH on patients with major depressive disorders. GBH has been widely used in East Asian countries without serious risk, and the risk associated with GBH in depressive patients is expected to be low.

### Objectives {7}

The current phase II randomised controlled trial explores the efficacy and safety of high-dose and low-dose GBH compared with placebo in 126 patients with MDD. The primary objective is to explore whether at least one of the two doses of GBH is effective compared with placebo in patients with MDD. At the post-treatment phase (week 8), the changes from baseline in the 17-item Hamilton Depression Rating Scale (HDRS) total score will be compared among three groups. Subsequent pairwise comparisons will be carried out between the two groups treated with low-dose GBH and placebo, and high-dose GBH and placebo. Also, based on the effect sizes of GBH compared with placebo, which will be examined in this phase II trial, the feasibility of the phase III therapeutic confirmative clinical trial will be established. The secondary objectives include analysis of the efficacy of low-dose and high-dose GBH in patients with MDD compared with placebo based on HDRS response, HDRS remission, and changes from baseline on the HDRS-6 subscale and Beck Depression Inventory-II (BDI-II) at the post-treatment (week 8). The safety of GBH compared with placebo will be assessed based on the incidence of intervention-related adverse events, measurement of vital signs, clinical and laboratory evaluations, and electrocardiograms (ECGs).

### Trial design {8}

This study will be a randomised, double-blind, placebo-controlled, and multi-arm parallel-group trial. Participants will be allocated in a 1:1:1 ratio to one of three parallel groups: high-dose GBH, low-dose GBH, and placebo. The HDRS-17 total scores in high-dose and low-dose GBH groups will be compared with those of the placebo group.

## Methods: participants, interventions, and outcomes

### Study setting {9}

This study is being conducted at three university hospitals in Korea specialising in Korean medicine. Two sites are located in Seoul and one site in Daejeon.

### Eligibility criteria {10}

#### Inclusion criteria

1. Men and women aged 19 to 65 years
2. Those diagnosed with major depressive disorder based on DSM-5 (Diagnostic and Statistical Manual of Mental Disorders-5) criteria
3. Those with a HDRS-17 total score of 18 or higher
4. Those who volunteered to participate and sign the agreement

#### Exclusion criteria

1. Participants with risks of suicide
2. Participants with major depressive disorder who need hospitalisation
3. Participants who received electroconvulsive therapy (ECT), vagal nerve stimulation (VNS), deep brain stimulation (DBS), light therapy, or transcranial magnetic stimulation (TMS) within 3 months prior to study
4. Participants who are diagnosed with and are being treated for panic disorder, obsessive disorder, post-traumatic stress disorder, or personality disorder
5. Participants with a history of manic, schizophrenic, or mixed episodes
6. Participants with current or lifetime alcohol or other substance abuse/dependence
7. Participants who are undergoing treatment with medicines or substances that may affect the degree of depression (e.g., anxiolytics, antidepressants, antipsychotics, corticosteroids, female hormones, L-dopa, digitalis, bromide, cyclosporin, disulfiram, isoniazid, and yohimbine) within 4 weeks
8. Participants who are undergoing taking antiplatelet therapy
9. Participants with a medical condition that may affect the degree of depression, including myocardial infarction, brain tumour, multiple sclerosis, pancreatic disease, hypo/hyperthyroidism, hyperparathyroidism, Addison’s disease, Cushing’s disease, rheumatoid arthritis, cancer, cerebrovascular disease, dementia, and epilepsy
10. Participants with chronic illnesses such as chronic active hepatitis, hypertension, and diabetes that are not well-controlled with appropriate treatment
11. Participants who are being treated for liver cancer, liver cirrhosis, chronic kidney failure, or congestive heart failure classified as NYHA class III–IV
12. Participants with liver dysfunction or renal impairment (based on ALT, AST, ALP ≥ twice the normal upper limit or creatinine > 2.0 mg/dL at screening)
13. Participants with hereditary conditions such as galactose intolerance, Lapp lactase deficiency, or glucose-galactose malabsorption
14. Participants carrying diseases that may affect the absorption of the drug or who manifest a digestive disorder after surgery
15. Participants who involved in other clinical trials within 1 month
16. Participants with hypersensitivity reactions and allergies to research drugs
17. Participants who are taking other herbal medicines
18. Participants who are unable to understand the consent form due to cognitive defects such as mental retardation or intellectual problems
19. Pregnant or lactating women
20. Participants who are likely to be pregnant and who do not agree to the approved methods of contraception (double contraception, intrauterine contraceptive, and spermicide) during the trial
21. Participants who are determined as unsuitable by the investigator

### Who will take informed consent? {26a}

The principal investigators, who are professors in the Department of Neuropsychiatry at Korean medicine hospitals, or sub-investigators, who are fellows or residents in the hospital and delegated by the principal investigators, will explain the clinical trial and the study drug in detail and seek informed consent for participation in the clinical trial. Participants will be informed that the effect of study drug is not validated, and placebo can be administered without active ingredient. Also, the standard treatment for patients with depressive disorders will be described in the informed consent form and explained by the investigators. Participants will be intensively educated to contact the investigators whenever they suspect severe depressive symptoms. The consent will be obtained prior to the screening process.

### Additional consent provisions for collection and use of participant data and biological specimens {26b}

Not applicable

### Interventions

#### Explanation for the choice of comparators {6b}

Placebo granules, which are identical to the experimental granules in shape, colour, scent, and taste and containing no active ingredients, will be used as comparators. Placebo granules will be produced and packaged by Hanpoong Pharm & Food Co., Ltd., (Jeonju, Korea) under good manufacturing practice (GMP) regulations of Korea. Placebo granules will consist of lactose hydrate, corn starch, hydroxypropyl methylcellulose, and caramel colour. Three placebo granule sachets (1.5 g for each, total 4.5 g) will be orally administered twice a day, for 8 weeks to participants in the placebo group.

#### Intervention description {11a}

GBH granules are produced and packaged by Hanpoong Pharm & Food Co., Ltd., (Jeonju, Korea) under good manufacturing practices (GMPs). GBH extract consists of *Cinnamomi Ramulus*, *Poria Sclerotium*, *Moutan Radicis Cortex*, *Persicae Semen*, and *Paeoniae Radix* (Table 1). A gramme of GBH granule contains 443.33 mg of GBH extract, lactose hydrate, and corn starch. Participants in the high-dose group will be administered three GBH granule sachets (1.5 g each, total 4.5 g) twice a day, for 8 weeks. Participants in the low-dose group will be treated with one GBH granule (1.5 g each, total 1.5 g) and two placebo granule sachets (1.5 g each, total 3 g) orally twice a day, for 8 weeks (Table 2). The high-dose GBH (GBH extract 3,900mg/day) is identical to the approved dose of GBH for another indication. Meanwhile, the low-dose GBH (GBH extract 1300mg/day) was calculated based on the human equivalent dose [20] converted from the optimal dose identified in a mouse model of depression.

**Table 1:** Ingredients and composition of GBH extract

Herbal name	Source species	Parts used	Composition ratio
Cinnamomi Ramulus	*Cinnamomum cassia* J. Presl	Branch	1
Poria Sclerotium	*Poria cocos* Wolf	Sclerotium	1
Moutan Radicis Cortex	*Paeonia suffruticosa* Andrews	Rhizodermis	1
Persicae Semen	*Prunus persica* Batsch	Seed	1
Paeoniae Radix	*Paeonia lactiflora* Pallas	Root	1

**Table 2:** Doses of GBH granules in three groups

Group	Daily dose	Daily dose of active ingredient
High-dose GBH	GBH granule 9 g	GBH extract 3900 mg
Low-dose GBH	GBH granule 3 g + placebo granule 6 g	GBH extract 1300 mg
Placebo	Placebo granule 9 g	None

#### Criteria for discontinuing or modifying allocated interventions {11b}

Participants are considered for treatment cessation and elimination from the study if they meet the following criteria.

1. Participants who do not meet inclusion and exclusion criteria
2. Participants with serious adverse events (SAEs)
3. Participants with adverse events warranting discontinuation of study drug
4. Participants with symptom aggravation and in need of another treatment
5. Participants who withdraw consent
6. Participants who are lost to follow-up
7. Participants whose compliance with treatment is below 70%
8. Participants who are unsuitable for continuing administration by the investigators

Additionally, procedures for assessing suicide risk of participants and discontinuing intervention in case of high suicide risk were established. During 8 weeks of treatment, the depressive symptoms of participants including suicide risk will be assessed every 2 weeks. Assessment of suicide risk will be carried out using the third item of HDRS and semi-structured interview for assessing suicide risk. High suicide risk will be determined by the researcher based on the presence of specific suicide ideation or suicide attempt. Participants with high suicide risk will be discontinued from the study immediately and transferred for appropriate management and treatment.

#### Strategies to improve adherence to interventions {11c}

To evaluate treatment adherence, the amount of drug return will be checked at every visit when participants visit the hospitals every 2 weeks during 8 weeks of administration. The investigator and clinical research pharmacist will determine the compliance of each participant and instruct them not to overdose.

#### Relevant concomitant care permitted or prohibited during the trial {11d}

##### Concomitant medication and care prohibited during the trial

1. Anxiolytics, antidepressants, antipsychotics, corticosteroids, female hormones, thrombolytic, blood coagulation inhibitor, L-dopa, Digitalis, bromide, cyclosporin, disulfiram, isoniazid, and yohimbine
2. Medications for treating or improving the symptoms of major depressive disorders
3. Medications that may affect the degree of depression
4. Other herbal medicines or dietary supplements
5. Non-pharmacologic interventions for improving depressive symptoms (e.g., acupuncture, relaxation therapy, electroconvulsive therapy (ECT), vagal nerve stimulation (VNS), deep brain stimulation (DBS), light therapy, or transcranial magnetic stimulation (TMS))

##### Concomitant medication permitted during the trial

Concomitant medications will be permitted based on the judgement of investigators, if the drugs are not prohibited during the trial and administered for more than 4 weeks during the screening period.

#### Provisions for post-trial care {30}

Follow-up observation will be performed at week 12, 4 weeks after the completion of the 8-week treatment. In case of adverse events, the investigator will evaluate the follow-up observations and provide the requisite treatment until the symptoms disappear.

#### Outcomes {12}

##### Primary outcome

The primary outcome is the change from the baseline HDRS-17 total score at week 8 (post-treatment). Baseline HDRS-17 total score and site will be used as the covariate. The change from the baseline in HDRS-17 total score at week 8 will be compared in groups treated with low-dose GBH and placebo, and high-dose GBH and placebo.

##### Secondary outcomes

1. Change from the baseline HDRS-6 subscale at week 8 (post-treatment)
2. HDRS response: proportion of participants with HDRS-17 total score reduced below 50% of the baseline score at week 8 (post-treatment)
3. HDRS remission: proportion of participants, whose HDRS-17 total score is less than 7 [21] at week 8 (post-treatment)
4. Change from the baseline BDI-II score at week 8 (post-treatment)
5. Change from the baseline Insomnia Severity Index (ISI) score at week 8 (post-treatment)
6. Change from the baseline anxiety state and anxiety trait in the State-Trait Anxiety Inventory YZ form (STAI-YZ) score at week 8 (post-treatment)
7. Change from the baseline anger state, anger trait, anger in, anger out, and anger control of State-Trait Anger Expression Inventory (STAXI) score at week 8 (post-treatment)
8. Change from the baseline 3-level version of EQ-5D (EQ-5D-3L) index at week 8 (post-treatment)

##### Exploratory outcomes

1. Korean Symptom checklist-95 (KSCL95) [22] will be measured at weeks 0, 4, 8, and 12. *T* scores of depression, anxiety, phobic anxiety, obsessive-compulsive disorder, post-traumatic stress disorder, aggressive state, somatisation, manic, paranoid ideation, schizophrenia, suicide, addiction, sleeping problems, and stress vulnerability will be calculated.
2. Pattern identification tool for depression (PIT-D) [23, 24] will be used at weeks 0, 4, 8, and 12. Pattern scores of stagnation of liver qi, deficiency of the heart and spleen, yin deficiency with excessive fire, qi deficiency with phlegm, and stagnation of qi with fire will be calculated.
3. Saliva melatonin and cortisol at 10 am will be measured at weeks 0, and 8.

### Participant timeline {13}


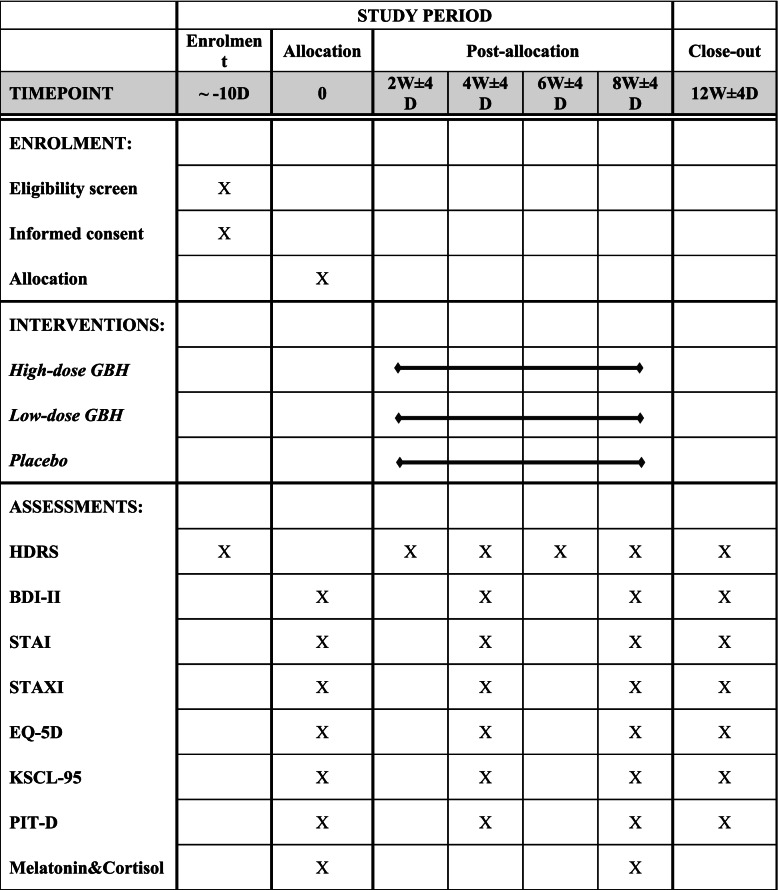


### Sample size {14}

Sample size was computed to detect the mean difference in change from the baseline HDRS-17 total score between placebo and low-dose GBH groups. The mean difference in scores between the two groups was assumed as four, which was reported to be the threshold points for minimal improvement in patients’ perspectives [25, 26]. Also, standard deviation was estimated to be 5.78, based on the results of a similar clinical trial of *Danzhiningshen* involving patients with depression [27]. The herbal composition of GBH and Danzhiningshen differs, but the two formulae contain *Moutan Radicis Cortex*. With a two-sided significance level of 5% and a power of 80%, 33.77 participants are required in each group of the trial. Considering the drop-out rate of 20%, 42 participants will be recruited per group, and a total of 126 will be investigated.

### Recruitment {15}

Participants will be recruited via notices on the hospital bulletin boards and online advertisements. Two sites in Seoul will recruit 45 participants each, and one site in Daejeon will recruit 36 participants. The number of participants recruited at each site can be changed as the recruitment progresses.

### Assignment of interventions: allocation

#### Sequence generation {16a}

Participants will be randomly assigned to either high-dose GBH, low-dose GBH, or placebo group in a 1:1:1 allocation ratio. A random number table for blocked randomisation will be designed for each site. The size of the block will be concealed.

#### Concealment mechanism {16b}

Participants will be sequentially numbered based on the enrollment. Allocation will be concealed, as the random number table is generated and maintained separately.

#### Implementation {16c}

The random number table for allocation will be generated by an independent statistician. The other investigators do not have access to the random number table. As the participants are enrolled in the trial, investigators will assign them a random number sequentially. Following the enrollment, participants will receive the study drug according to the assigned random number. The study drugs are packaged identically and contain either high-dose GBH, low-dose GBH, or placebo granule according to the random number table. Thus, the randomisation will be conducted without any knowledge of the principal investigators or sub-investigators.

### Assignment of interventions: blinding

#### Who will be blinded {17a}

Trial participants and investigators will be blinded. Participants will not know which study drug (high-dose GBH, low-dose GBH, or placebo granule) they are treated with. High-dose GBH, low-dose GBH, and placebo granules will be packaged identically, including the shape, colour, scent, and taste. The principal investigators, sub-investigators, clinical research coordinator, and clinical research pharmacist will be blinded to the group assignment of each participant.

#### Procedure for unblinding if needed {17b}

In case of serious medical emergencies, the code can be revealed under exceptional circumstance to determine the participant allocation. Based on the decision of the principal investigators or sub-investigators to unmask the identity of the participant, the principal investigator will hold an investigators’ meeting. If it is determined that it is necessary to unmask, the principal investigator will request breaking the participant code in writing, and the person in charge of managing random number sequence will notify the participant’s allocation information.

### Data collection and management

#### Plans for assessment and collection of outcomes {18a}

##### Primary outcome

HDRS is a representative clinician rating scale for depression. It consists of 17 items measuring the severity of depressive symptoms, and the HDRS-17 total scores range from 0 to 52, with the higher score indicating severe symptoms [28, 29]. The Korean version of HDRS validated in 2005 will be used in this study [30]. HDRS will be evaluated by trained investigators working as fellows or residents at the Department of Neuropsychiatry in Korean medicine. To ensure the consistency of HDRS, the investigators from three sites met to conduct training and discussion sessions before the initiation of clinical trial. The HDRS assessment will be carried out according to the Structured Interview Guide for the Hamilton Depression Rating Scale (SIGH-D) [31]. HDRS will be measured every 2 weeks during the 8 weeks of administration (week 0, 2, 4, 6, 8) and follow-up assessment (week 12).

##### Secondary outcomes

The HDRS-6 subscale consists of 6 items in HDRS, including depressed mood, feelings of guilt, work and activities, psychomotor retardation, psychic anxiety, and general somatic symptoms [32]. It can be used as a unidimensional measure of depressive severity, excluding insomnia and gastrointestinal symptoms [33, 34]. BDI-II, ISI, STAI, STAXI, and EQ-5D-3L are all validated self-reporting questionnaires. BDI-II is one of the patient self-rating scales for depression. It consists of 21 items measuring the severity of depressive symptoms, ranging from 0 to 63 [35]. The Korean version of BDI-II was validated in 2011 and will be used in this study [36]. ISI will be used to measure the severity of insomnia. ISI is a self-rating scale for insomnia, consisting of 7 items, and ranges from 0 to 28 [37]. The Korean version of ISI will be used in this study [38]. STAI-YZ will be used to measure the severity of anxiety symptoms [39, 40]. STAXI will be used to measure the severity of anger symptoms [41, 42]. EQ-5D-3L will be used to measure the quality of life. A certified Korean version of EQ-5D-3L will be used in this study [43, 44]. BDI-II, ISI, STAI, STAXI, and EQ-5D-3L will be measured every 4 weeks during the 8 weeks of administration (week 0, 4, 8) and follow-up assessment (week 12).

#### Plans to promote participant retention and complete follow-up {18b}

At every visit, the date of next visit will be scheduled, and the participant will be notified the day before the date of visit. The questionnaires will be checked for missing values and the participant will be requested to answer all the items listed therein.

#### Data management {19}

Data will be collected using an electronic case report form (eCRF) in the Electronic Data Capture (EDC) system of CRScube Inc. The eCRF will be designed according to the study protocol, and the data validation specification will be completed before the trial initiation. After the e-CRF release, the system query will be evaluated regularly (bi-monthly). Medical coding of adverse events and medical history will be entered using the Medical Dictionary for Regulatory Activities (MedDRA) [45]. The database will be locked following the completion of all data entry, query resolution, and medical coding procedures.

#### Confidentiality {27}

Records of participants’ personal information will be managed in accordance with relevant regulations to maintain confidentiality. The eCRF will be recorded and classified based on the participants’ identification code, and not the participant’s name. However, the clinical research associates (CRAs) who are in charge of monitoring, along with the auditor, the Institutional Review Board (IRB), and the director of Ministry of Food and Drug Safety (MFDS), may access the medical records and other source documents to verify the reliability of data in this clinical trial. The individuals or institutions with direct access to the data related to the trial have a duty of confidentiality regarding participants’ personal information. When the results of the clinical trial are published, the records used to identify the participants will remain confidential.

#### Plans for collection, laboratory evaluation, and storage of biological specimens for genetic or molecular analysis in this trial/future use {33}

None

### Statistical methods

#### Statistical methods for primary and secondary outcomes {20a}

##### Primary outcome

The change from the baseline in HDRS-17 total score at week 8 (post-treatment) among the three groups will be compared via analysis of covariance (ANCOVA). The group will be used as the fixed factor, and baseline HDRS-17 total score and site will be used as the covariate. The effect of group on HDRS-17 total score after adjusting for baseline score and site will be calculated. Subsequent pairwise comparisons will be performed with low-dose GBH vs placebo and high-dose GBH vs placebo using Dunnett’s test. The least square mean and standard error of three groups, and the estimated mean difference (95% CI) and adjusted *p*-value compared with placebo group will be presented.

##### Secondary outcomes

Analyses of continuous variables of HDRS-6 subscale, BDI-II, ISI, STAXI, and EQ-5D-3L index at week 8 (post-treatment) will be performed using the same method described for HDRS-17 total score.

The proportion of HDRS response and remission at week 8 (post-treatment) in the three groups will be compared via logistic regression analysis. The group, baseline HDRS-17 total score, and site will be used as the predictors. The estimated odds ratio (95% CI) and *p*-value with the placebo group as the reference will be presented.

#### Interim analyses {21b}

There are no interim analyses planned.

#### Methods for additional analyses (e.g., subgroup analyses) {20b}

##### Additional analyses using repeated measures

The Linear Mixed Model (LMM) will be used to analyse the effect of group on continuous variables (HDRS-17 total score, HDRS-6 subscale, BDI-II, ISI, STAXI, and EQ-5D index) over time. Also, the generalised estimating equations (GEE) analysis will be used to analyse the effect of group on binary variables (HDRS response and remission) over time.

##### Additional adjusted analyses

Primary and secondary analyses will be conducted with the baseline score and the site as the covariates. Additional analyses will be performed via ANCOVA of primary and secondary outcomes at the post-treatment (week 8) with age, sex, site, and baseline score as covariates.

##### Subgroup analyses

Subgroup analyses will be conducted based on the sex and baseline KM pattern.

#### Methods in analysis to handle protocol non-adherence and any statistical methods to handle missing data {20c}

##### Definition of analysis population

Full analysis set will include all randomised participants, except as follows:

1. Participants who are found to be ineligible for the trial after randomisation
2. Participants who have never been exposed to the investigational product
3. Participants who have never been assessed after randomisation

Per protocol set will include participants who have completed the trial process without violating the protocol. The following participants will be excluded from the per protocol set.

1. Participants who dropped out from the trial
2. Participants who are found to be ineligible for the trial
3. Participants with less than 75% compliance with the study treatment protocol
4. Participants who are considered as a major violation for any other reason

Safety set will include all participants who have received the intervention at least once. Participants who have never been treated with the investigational product will be excluded from the safety set.

For the efficacy outcome analysis, the full analysis set will be mainly analysed, and per protocol set will be additionally analysed. A safety set will be used for the safety analysis. Multiple imputations will be performed to handle missing data.

#### Plans to give access to the full protocol, participant-level data, and statistical code {31c}

The datasets used and/or analysed during the current study can be made available by the corresponding author upon reasonable request in accordance with the research collaboration and data transfer guidelines of the Korea Institute of Oriental Medicine.

### Oversight and monitoring

#### Composition of the coordinating Centre and trial steering committee {5d}

A contract research organisation (CRO) is in charge of monitoring, data management, and statistical analysis. Also, the pharmaceutical company will produce and manage the investigational product. The Korea Institute of Oriental Medicine (KIOM) is responsible for planning of the trial, managing overall trial progress, MFDS approval, and reporting. The routine monitoring visits will be conducted 42 times in total. Compliance with protocol and good clinical practices will be monitored, in addition to full source data verification.

#### Composition of the data monitoring committee, its role, and reporting structure {21a}

No data monitoring committee was established for this trial, because the trial represents a phase II therapeutic exploratory study.

#### Adverse event reporting and harms {22}

All adverse events reported by the participants or observed by the investigators will be recorded. The severity of adverse events and causality attributed to the study drug will be evaluated.

#### Frequency and plans for auditing trial conduct {23}

Audit will be conducted twice, including at one site in Seoul and another site in Daejeon. Independent auditor working at KIOM will conduct the audit.

#### Plans for communicating important protocol amendments to relevant parties (e.g., trial participants, ethical committees) {25}

If it is necessary to revise the protocol, the principal investigators must seek the approval of IRB. For every revision, protocol agreement should be signed by the principal investigators and sponsors.

#### Dissemination plans {31a}

Results of this clinical trial will be disclosed completely in international peer-reviewed journals. Both positive and negative results will be reported.

## Discussion

This randomised controlled trial is designed to investigate the safety and efficacy of GBH compared with placebo, in patients with major depressive disorders. Also, this trial is designed to determine the optimal dose of GBH for major depressive disorders, for further investigation in a phase III trial.

GBH granule is one of the products of herbal medicine manufactured by various pharmaceutical companies according to the Korean Herbal Pharmacopoeia (KHP) and following good manufacturing practice (GMP) guidelines. GBH is approved by the Korean MFDS for the following indications: dysmenorrhea, menstrual cramps, menopausal disorders, dizziness, and bruise. In case of approved herbal medicines with the target indication out of range for the indications previously specified, an investigational new drug (IND) application for a new indication is required in Korea [46]. Depressive disorder was not one of the previously approved indications for GBH. This trial protocol was approved by Korean MFDS in October 2018, and the trial was initiated in December 2019.

Various clinical trials have been conducted to explore the efficacy of herbal medicines for depression [47–49]. Previous randomised trials mostly included patients with mild-to-moderate depression [47]. In this trial, we plan to enrol patients with moderate depression whose HDRS-17 total score is 18 or higher. Also, patients who are undergoing treatment with antidepressants will be excluded from the trial, and the efficacy and safety of GBH monotherapy compared to placebo will be examined. Intensive and close monitoring of suicide risk factors will be conducted to prevent and minimise the risk.

The novel herbal composition of GBH is designed for patients with depression. In previous randomised trials of depression, *Bupleuri Radix*, *Angelicae Sinensis Radix*, *Paeoniae Radix*, and *Poria Sclerotium* were frequently used [47]. GBH also contains *Poria Sclerotium* and *Paeoniae Radix*. The other herbal components of GBH include *Cinnamomi Ramulus*, *Moutan Radicis Cortex*, and *Persicae Semen*. The composition of GBH also differs from the frequently used prescription pattern recommended for depressive disorders in Taiwan [50]. Based on the results of in vivo studies of GBH, amelioration of depression is expected.

This trial will be conducted in three arms: high-dose GBH, low-dose GBH, and placebo. Few clinical trials of herbal medicine involved two different doses. Another goal of this trial is to investigate the dose of GBH for the next phase III therapeutic confirmatory trial. The two doses of GBH in this therapeutic exploratory trial were set based on the dose used in routine clinical practice (high-dose) and the dose based on animal studies (low-dose). The antidepressive effect of low-dose GBH is also expected as it represents the human equivalent [20] of the optimal dose identified in animal studies.

The primary outcome of this trial is the HDRS-17 total score. It is a representative clinician-rating scale designed to measure the severity of depressions [51]. Korean MFDS recommended HDRS-17 total score [29] or Montgomery Asberg Depression Scale (MADRS) [52] as the primary outcome in clinical trials of antidepressants [53]. Also, most previous clinical trials of herbal medicine for depression used HDRS-17 total score [47, 54, 55]. However, few critics suggest that HDRS is a multidimensional rating scale and the cumulative 17-item HDRS score has a limited role in reflecting the core symptoms of depression [33, 34, 56]. The 17 items of HDRS also include multiple components like insomnia and gastrointestinal and genital symptoms. The HDRS-6 subscale has been suggested to measure the core symptoms of depression such as depressed mood and feelings of guilt [32]. The HDRS-6 subscale will represent one of the secondary outcomes in this trial. HDRS will be used every 2 weeks during the 8 weeks of administration in this trial. Based on the results of preclinical studies, the pharmacological mechanism of GBH appears to involve glutamate modulation via NMDA receptor, as well as downstream activation of BDNF and CREB signalling pathway [15]. Glutamate receptor antagonists have been reported to exhibit a potential fast-acting therapeutic effect in alleviating depressive symptoms [57]. In this clinical trial, the rapid pharmacologic effect of GBH on depressive symptoms will also be investigated exploratory.

## Trial status

Recruiting for the trial started in December 2019. The current protocol represents version 2.8 of 28-5-2020. Patient recruitment is estimated to be completed around May 2022.

## Data Availability

The datasets used and/or analysed in the current study will be made available from the corresponding author upon reasonable request.

## Abbreviations

ANCOVA: Analysis of covariance
ANOVA: Analysis of variance
BDI-II: Beck Depression Inventory-II
CRA: Clinical research associate
CRIS: Clinical Research Information Service
CRO: Contract research organisation
DBS: Deep brain stimulation
DSM-V: Diagnostic and Statistical Manual of Mental Disorders-5
ECG: Electrocardiogram
ECT: Electroconvulsive therapy
eCRF: Electronic case report form
EDC: Electronic Data Capture
GBH: *Gyejibokryeong-hwan*
GMP: Good manufacturing practice
HDRS: Hamilton Depression Rating Scale
IND: Investigational new drug
IRB: Institutional Review Board
ISI: Insomnia Severity Index
KHP: Korean Herbal Pharmacopoeia
KSCL95: Korean Symptom Checklist-95
MDD: Major depressive disorder
MedDRA: Medical Dictionary for Regulatory Activities
MFDS: Ministry of Food and Drug Safety
OECD: Organisation for Economic Cooperation and Development
PIT-D: Pattern identification tool for depression
RCT: Randomised controlled trial
SAE: Serious adverse event
SIGH-D: Structured Interview Guide for the Hamilton Depression Rating Scale
STAI-YZ: State-Trait Anxiety Inventory YZ form
STAXI: State-Trait Anger Expression Inventory
TEAM: Traditional East Asian medicine
TMS: Transcranial magnetic stimulation
VNS: Vagal nerve stimulation
